# The role of nuclear localization signal in parvovirus life cycle

**DOI:** 10.1186/s12985-017-0745-1

**Published:** 2017-04-14

**Authors:** Peng Liu, Shun Chen, Mingshu Wang, Anchun Cheng

**Affiliations:** 1 Institute of Preventive Veterinary Medicine, Sichuan Agricultural University, No. 211 Huimin Road, Wenjiang District, Chengdu, Sichuan 611130 China; 2 Research Center of Avian Disease, College of Veterinary Medicine of Sichuan Agricultural University, Chengdu, Sichuan 611130 China; 3 Key Laboratory of Animal Disease and Human Health of Sichuan Province, Chengdu, Sichuan 611130 China

**Keywords:** Parvovirus, Nuclear localization signal, Nuclear transport

## Abstract

Parvoviruses are small, non-enveloped viruses with an approximately 5.0 kb, single-stranded DNA genome. Usually, the parvovirus capsid gene contains one or more nuclear localization signals (NLSs), which are required for guiding the virus particle into the nucleus through the nuclear pore. However, several classical NLSs (cNLSs) and non-classical NLSs (ncNLSs) have been identified in non-structural genes, and the ncNLSs can also target non-structural proteins into the nucleus. In this review, we have summarized recent research findings on parvovirus NLSs. The capsid protein of the adeno-associated virus has four potential nuclear localization sequences, named basic region 1 (BR), BR2, BR3 and BR4. BR3 was identified as an NLS by fusing it with green fluorescent protein. Moreover, BR3 and BR4 are required for infectivity and virion assembly. In *Protoparvovirus*, the canine parvovirus has a common cNLS located in the VP1 unique region, similar to parvovirus minute virus of mice (MVM) and porcine parvovirus. Moreover, an ncNLS is found in the C-terminal region of MVM VP1/2. Parvovirus B19 also contains an ncNLS in the C-terminal region of VP1/2, which is essential for the nuclear transport of VP1/VP2. Approximately 1 or 2 cNLSs and 1 ncNLS have been reported in the non-structural protein of bocaviruses. Understanding the role of the NLS in the process of parvovirus infection and its mechanism of nuclear transport will contribute to the development of therapeutic vaccines and novel antiviral medicines.

## Background

Parvoviruses are the smallest animal DNA viruses and constitute a widely dispersed virus family. They infect a wide variety of hosts, including insects, birds, and mammals. The family *Parvoviridae* is divided into two subfamilies, the *Parvovirinae* and the *Densovirinae*. The former infects vertebrates, while the latter infects invertebrates. Through phylogenetic analyses based on DNA and protein sequences, the subfamily *Parvovirinae* has been divided into eight genera: *Amdoparvovirus, Aveparvovirus, Bocaparvovirus, Copiparvovirus, Dependoparvovirus, Erythroparvovirus, Protoparvovirus,* and *Tetraparvovirus*. Human parvovirus B19, belonging to the genus *Erythroparvovirus*, is a prominent human pathogen and causes severe syndromes in pregnant women and in individuals with underlying immune or haematologic disorders, such as hydrops foetalis and arthropathy [1]. The adeno-associated virus (AAV), a member of *Dependoparvovirus*, is non-pathogenic, and its infection and replication depend on helper viruses, such as adenoviruses [2]. Moreover, AAV is a vital gene therapy vector for human application. The bocavirus is classified in the genus *Bocaparvovirus* and can be detected in humans [3], cattle [4, 5], and other species. The human bocavirus is prevalent among children and can cause respiratory tract infections [6]. In animals, several parvoviruses, such as goose parvovirus [7], canine parvovirus [8], and bovine parvovirus [9], can cause enteritis and diarrhoea.

*Parvoviridae* is a family of 5-kb single-stranded DNA viruses with a non-enveloped capsid [10]. At both terminals, the linear, single-stranded genome contains palindromic sequences forming inverted terminal repeats, which can form a hairpin structure. The inverted terminal repeats provide most of the *cis-*acting information, which is required for both viral DNA replication and encapsidation [11]. There are two open reading frames (ORFs) in the viral genome. The left ORF, ORF1, encodes non-structural (NS) proteins, while the right ORF, ORF2, encodes two or three viral structural proteins. However, the *Bocaparvovirus* has an extra ORF located between ORF1 and ORF2 that encodes the non-structural protein (NP) 1 [12, 13]. The NS protein is a replicate protein needed for viral genomic replication [14] and works as a cytotoxic protein leading to apoptosis of host cells [15]. Generally, the virion is composed of two or three capsid proteins (VP1-VP3) which share an identical C-terminal sequence. The VP1 sequence comprises the entire VP2 sequence and an ~140 amino acid N-terminal extension called VP1 unique region (VP1u). The VP1u, especially the phospholipase A_2_ (PLA_2_) domain and the nuclear localization signal (NLS) located in the unique N terminus of VP1, is necessary for infectivity [16]. The PLA_2_ domain is required for parvovirus infectivity [17, 18], while the NLS plays an important role in the parvovirus replication cycle [19, 20]. In the early steps of infection, the NLS assists the translocation of viral genomes to the nucleus, whereas in the late steps of infection, the NLS is required for nuclear transport of viral capsid proteins.

NLSs are usually composed of basic residues (K and R), tend to be hydrophilic, and are divided into classical (cNLS) and non-classical (ncNLS) types. The cNLSs are further divided into classical monopartite NLS and classical bipartite NLS. In the cytoplasm, the small proteins can be transported into the nucleus through the nuclear pore complex freely or by passive diffusion, while the larger proteins (>50 kDa) require active transport. Large proteins can be transported into the nucleus with the help of an NLS [21]. Notably, the functions of NLSs have been identified in some DNA viruses, such as herpesvirus [22, 23], circovirus [24, 25], as well as some RNA viruses [26, 27]. An NLS identified in the herpesvirus VP1/2 capsid protein is important for infection via capsid routing to the nuclear pore complex [22]. Furthermore, an NLS found in the polymerase of the borna disease virus is essential for targeting this polymerase into the nucleus [26]. Several ncNLSs and cNLSs, which can guide the capsid protein into the nucleus and play a role in virus infection, have been identified in the structural proteins of parvoviruses. In this paper, based on the latest research progresses, we summarize the current knowledge about the role of NLSs in the parvovirus replication cycle, which may help us to better understand the molecular pathogenesis of parvoviruses.

## The NLS of parvoviruses

### The NLS of *Dependoparvovirus*

**Fig. 1:**
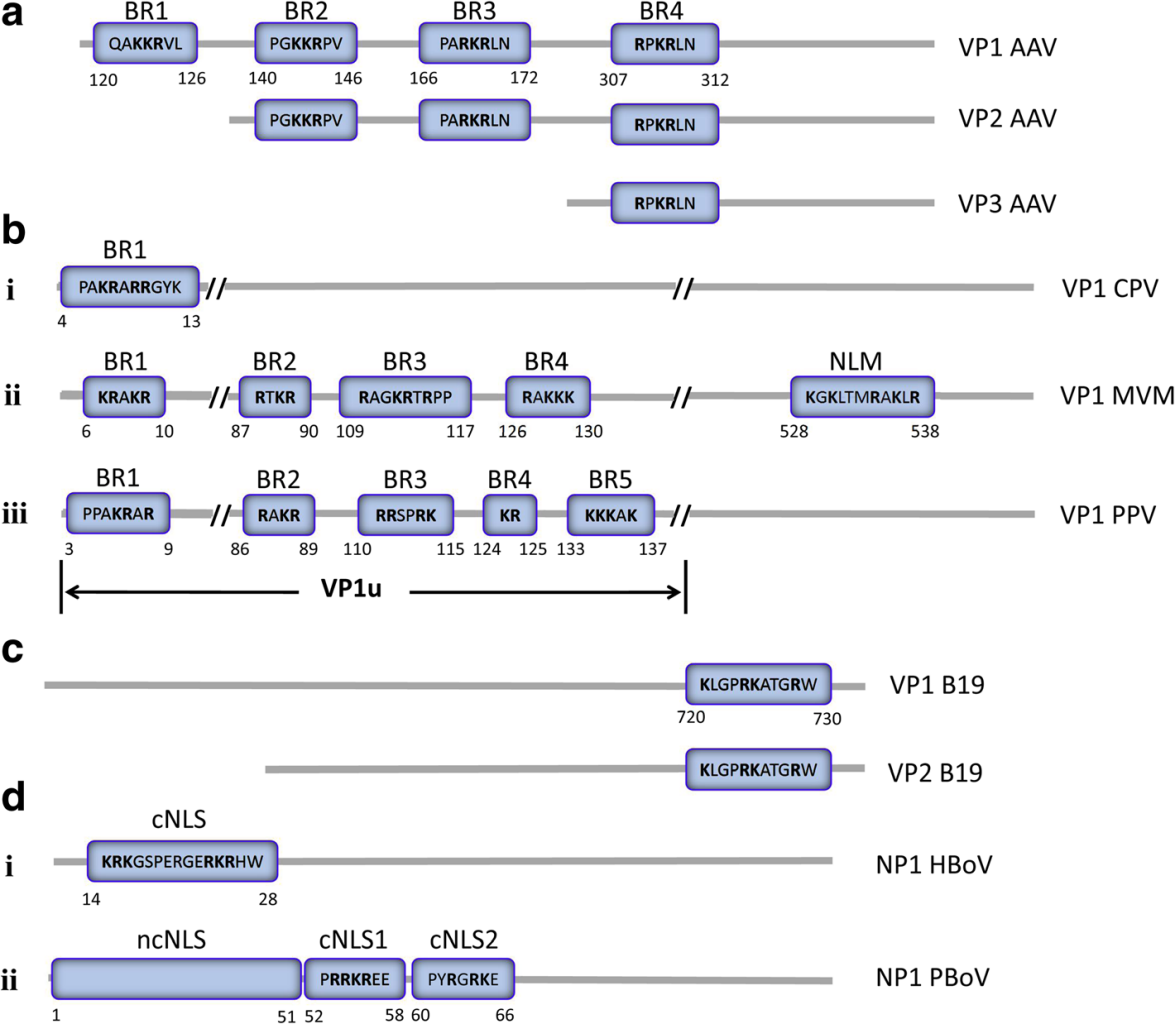
A schematic picture of parvovirus NLSs. **a** The BR1, BR2, and BR4 are potential NLSs that need further identification. BR3 was identified as an NLS. **b** *i*: BR1 was identified as a cNLS in the VP1u of CPV. *ii*: BR1 and BR2 were identified as a cNLS and a weak NLS respectively in the VP1u of MVM, and one ncNLS was identified in the C-terminus of MVM. *iii*: BR1 is a classical monopartite NLS, BR4-5 is classical bipartite NLS, and BR2 and BR3 are weak NLSs in the VP1u of PPV. **c** Human parvovirus B19 contains one ncNLS in the C-terminus. **d** *i*: HBoV has a classical bipartite NLS and one ncNLS in the N-terminus of NP1. *ii*: PBoV has two classical NLSs and one ncNLS in the N-terminus of NP1

Four basic regions (BRs), BR1, BR2, BR3, and BR4, were found in the capsid protein of AAV by using the PSORT II program (Fig. 1a). BR1 (120QAKKRVL126) is located in the VP1 N-terminus of AAV. BR2 (140PGKKRPV146) and BR3 (166PARKRLN172) are found in the overlapping sequence region of VP1 and VP2. BR4 (307RPKRLN321) is located in the overlapping sequence region of VP1, VP2, and VP3. BR1 and BR2 were found to have minor effects on viral infectivity [28]. Michael Ruffing *et al.* showed that both AAV VP1 and VP2 capsid proteins were localized in the nucleus of HeLa cells when they were transfected with a signal plasmid, which may be because BR2, BR3, and BR4 are encoded by overlapping sequences of VP1 and VP2 [29]. This indicated that BR1 is not necessary for VP localization to the nucleus. However, in a previous alanine scanning mutagenesis study on the AAV2 capsid, the AAV2 with mutagenesis of BR1 had low infectivity, showing viral titres that were 1 to 3 logs lower than the wild-type titres [30]. Moreover, Joshua C. Greiger *et al*. found that mutagenesis of BR1 and BR2 affected AAV2 infectivity by 4-fold and 10-fold, respectively, and hence, these BRs played an important role in virus infectivity [28]. BR3 is essential for localization of VP2 into the nucleus and for virion infectivity. In another study, Mainul Hoque *et al*. transfected cells with a series of VP2 deletion mutants and identified a nuclear localization sequence in the VP2 N-terminus of AAV2 (denoted BR3), which was necessary for leading VP2 to the nucleus [20]. The authors concluded that this NLS is necessary for translocation of the capsid protein from the cytoplasm to the nucleus prior to virion assembly [20]. Furthermore, the expression of AAV VP3 alone was distributed between the nucleus and cytoplasm, whereas co-expression of VP3 with other structural proteins led to its nuclear localization, which suggests that VP1 or VP2 (BR2 or BR3) is required for accumulation of VP3 in the nucleus [29]. However, Joshua C. Greiger *et al*. demonstrated that BR3 is not essential for the accumulation of capsid proteins during virion production but is needed for targeting and infectivity of the virion [28]. BR4 may be essential for virion assembly. When BR4 was mutagenized, there were no intact virion particles in the nucleus. However, capsid proteins with mutant BR4 were detected in the nucleus [28]. This result indicated that the right sequence in BR4 of AAV2 is important for the assembly of intact virions in the nucleus. Together, each BR plays a different role in the viral infection process. Moreover, whether the BR1, BR2, and BR4 have the ability for nuclear localization should be the subject of further study.

### The NLS of *Protoparvovirus*

A BR of the canine parvovirus (CPV) capsid protein sequences has been analysed, and it has basic amino acids (K and R) common with most of the previously identified nuclear localization signals (Fig. 1b i). Based on that information, one peptide (PAKRARRGYKC) of the BR1 was synthesized and subsequently was conjugated with bovine serum albumin. The conjugates were labelled with rhodamine B and microinjected into the cytoplasm of A72 cells. Notably, the BR1 containing the N-terminal region (aa 4PAKRARRGYK13) of CPV was capable of efficiently transporting bovine serum albumin into the nucleus of A72 cells at 60 min post-injection [31]. Therefore, the BR1 (4PAKRARRGYK13) located in the CPV VP1 capsid protein was identified as having NLS functions. Another study using glycine scanning mutagenesis indicated that lysine at position 6 and two arginines at positions 7 and 9 of the VP played important roles in its nuclear targeting activity [32]. Subsequently, an infectious clone of CPV was constructed to generate mutated recombinant virus. When Lys 6, Arg 7, and Arg 9 of the CPV VP were substituted by Ala, the infectivity of the virus was diminished. Therefore, the BR1 is essential for viral infectivity. Interestingly, the infectivity of virus was not affected by single substitution of either Lys 6 or Arg 7 of CPV with Ala, showing titre levels similar to the wild-type CPV [31]. Although the motif 4PAKRARRGYK13 was identified as the NLS of CPV, its role in the early steps of infection when viruses deliver their genomes into the nucleus for replication and transcription, as well as in the late steps of infection when progeny viral capsid subunits are transported into the nucleus for assembly into progeny virions, was largely unknown.

A nuclear localization motif (NLM) and four BRs were found in the capsid protein of parvovirus minute virus of mice (MVM). The NLM is located in the overlap region of VP1/VP2, while the BRs are found in the N-terminus of VP1 [19, 33]. Moreover, a bipartite cNLS, which was found in the position 194 to the 216 of NS1 protein gene (194KK[X]_18_KKK216), is essential for translocation of NS1 into the nucleus [34]. The NLM (528KGKLTMRAKLR538) is composed of five basic amino acids (K and R) in the C-terminal residues of VP1/VP2 and located in a beta-sheet (Fig. 1b ii). The four BRs, called BR1 (6KRAKR10), BR2 (87RTKR90), BR3 (109RAGKRTRPP117), and BR4 (126RAKKK130), are highly conserved among parvoviruses (Fig. 1b ii). Different BRs play different roles in viral protein localization [19, 33]. By constructing a series of contiguous and overlapping deletion mutants, two major nuclear localization regions, BR1 and NLM, were confirmed as being involved in the accumulation of VP1 in the nucleus of infected cells. However, the MVM BR2 behaved as a weak NLS, while BR3 and BR4 lacked NLS activity [19]. In addition, the NLM is necessary for the nuclear localization of MVM VP2 protein and displays nuclear localization activity for VP subunits. The nuclear location of MVM-VP plasmid with the NLM and without BRs was identified, suggesting that the NLM plays an important role in the late steps of infection leading to progeny viral capsid subunits entering the nucleus [19, 33]. Moreover, NB324K cells were transfected with an infectious MVM plasmid with all BR sequences mutated or deleted, and the result suggested that all BR sequences were not essential for the nuclear translocation of the progeny viral capsid subunits [19, 33]. However, the mutant virions showed lower infectivity than WT virions, which indicated that the BR sequences may play an important role in the early steps of the infection process. Considering the PLA_2_ domain is located between BR1 and BR2, a mutation or deletion in BR1 and BR2 may have an influence on the activity of phospholipase. Thus, the specific function of the BR sequence should be further studied.

Five BRs were found in the VP1u of porcine parvovirus (PPV), named BR1 (3PPAKRAR9), BR2 (86RAKR89), BR3 (110RRSPRK115), BR4 (124KR125), and BR5 (133KKKAK137) [35] (Fig. 1b iii). Both BR1 and the combination of BR4 and BR5 (BR4-5) were cNLSs, and the BR4-5 was a bipartite NLS. BR1 and BR4-5 were required for the onset of infection [35]. To investigate the NLS activity of PPV BRs, each of the BRs with EGFP tag was expressed in eukaryotic porcine fibroblast cells. EGFP-BR1 and EGPF-BR4-5 fused proteins were almost exclusively localized in the nucleus of cells, indicating the NLS activity of the BR1 and BR4-5. However, the EGFP-BR2 and EGFP-BR3 were only slightly accumulated in the nucleus of cells. Furthermore, a recombinant PPV infection clone, with the PPAKRAR sequence of BR1 and the KKKAK of BR4-5 substituted by neutral hydrophilic polar amino acids (such as Asn, Thr, and Gln), was constructed and used to transfect porcine fibroblast cells. Consequently, no infectious virus was rescued [35]. Interestingly, the infectious virions, which had lower infectivity than WT virus, could be rescued when the infection clone had only BR1 or BR4-5 mutated [35]. These results indicate that the BR1 and BR4-5 play an important role in the PPV infectious cycle. Moreover, three potential regions of NLM were identified in the capsid of PPV, including an external region (containing of R374, R393, and R565) on the surface of the assembled capsid, a central region (containing of K475, R477) in the interior region of the trimer, and an internal region (containing of K272, K275, K487, R533, K535, R576) in the interior face of the capsid [35]. Mutation experiments confirmed that K275 and R533 were important for nuclear targeting of PPV VP2. Any mutagenesis of single amino acid at K275 or R533 was able to block the transport of VP2 into the nucleus, which indicates that the internal region is the NLM of PPV. Similar to the NLM of MVM, the NLM of PPV is needed for the correct folding of the protein and is responsible for translocation of VP2 into the nucleus. Collectively, the BR1 and BR4-5, located in the VP1 N-terminus of PPV, can assist the localization of the virus to the nucleus during the early steps of infection after the exposure of the VP1 N-terminus or at the late steps of infection when the capsid subunits are transported to the nucleus. However, the functions of NLSs and potential NLMs of PPV in the infection process need to be further illustrated.

### The NLS of *Erythroparvovirus*

An ncNLS was identified and located in the C-terminal region of the human parvovirus B19 major capsid protein VP2 (Fig. 1c). The sequence, 720KLGPRKATGRW730, is necessary for nuclear targeting of VP2 [36]. It is conserved among the C-terminal region of erythrovirus VP2 proteins. Interestingly, no cNLS was predicted in B19 VP2 capsid protein by using the PSORT II program. The major capsid protein VP2 of parvovirus B19 was located in the nucleus of primary erythroid cells by using indirect immunofluorescence analysis [36]. To find the nuclear localization signal of B19 VP2 capsid protein, the localization of full-length VP2 (aa VP 228-781) protein and a C-terminal, truncated form of VP2 (aa VP 228-670) were analysed by using indirect immunofluorescence, which showed that the C-terminal, truncated VP2 protein aberrantly remained in the cytoplasm of transfected COS-6 cells. Subsequently, three clusters of basic residues included between amino acid 720 and 781 were found. Then, the KLGPRKATGRW sequence (aa VP 720-730) was fused into pEGFP-C1, and the fluorescence signal was detected in the nucleus of transfected COS-6 cells [36]. Further experiments indicated that this sequence is also capable of transporting β-galactosidase fusion protein into the nucleus, confirming its importance in nuclear transport [36]. Although its function has been identified, the role of this sequence in the replicative cycle of erythroviruses is still unclear and thus, should be confirmed by constructing a B19 infection clone.

### The NLS of *Bocaparvovirus*

The non-structural protein (NP) 1 of human bocavirus (HBoV), which is encoded by the third open reading frame, is critical for the expression of the viral capsid protein. Jianming Qiu *et al.* found that NP1 is required for maturation of the capsid protein-encoding mRNA [37]. However, other non-structural proteins are not necessary for the expression of HBoV VP1/VP2/VP3. A classical bipartite NLS (cNLS aa 14–28) and an ncNLS (aa 7–50) were found in the N-terminal region of the NP1 gene (Fig. 1d i), both of which can transport a heterologous large cytoplasmic protein, β-galactosidase fusion protein, into the nucleus [38]. Moreover, two cNLSs (cNLS1 aa 52–58 and cNLS2 aa 60–66) and an ncNLS (aa 1–51) were identified in the N-terminal region of porcine bocavirus (PBoV) NP1 protein [39] (Fig. 1d ii). Amongst these, the two cNLSs have stronger nuclear transport ability than the ncNLS. Notably, this N-terminal region can inactivate the promoter activity of interferon β [39]. Although the NLSs were identified in NP1 of HBoV and PBoV, whether the capsid proteins also contain the NLS is unknown and remains to be studied.

## Conclusions

Parvoviruses are DNA viruses, which bind to the receptor of host cell surface upon infection and are subsequently internalized into the cytoplasm, following which they get into the nucleus with the guidance of the NLS and replicate and assemble in the nucleus of host cells. In the process of infection, the NLS is required for the virion life cycle, especially for the nuclear import of progeny viral proteins. In this review, we summarized the roles of the NLS in the parvovirus life cycle. However, the interaction between the functional NLS of parvovirus and importin of the host is still unknown. Accordingly, a thorough understanding of the mechanisms of the interplay between the NLS of parvovirus and the importin of the host may contribute to the development of antiviral vaccine candidates and novel inhibitors.

## Abbreviations

AAV: Adeno-associated virus
B19: Human parvovirus B19
BR: Basic region
cNLS: Classical nuclear localization signal
CPV: Canine parvovirus
HBoV: Human bocavirus
MVM: Minute virus of mice
ncNLS: Non-classical nuclear localization signal
NLM: Nuclear localization motif
NLS: Nuclear localization signal
NP: Non-structural protein
NS: Non-structural
PBoV: Porcine bocavirus
PLA_2_: Phospholipase A_2_
PPV: Porcine parvovirus
VP1u: VP1 unique region

## References

[1] Allander T, Jartti T, Gupta S, Niesters HGM, Lehtinen P, Osterback R, Vuorinen T, Waris M, Bjerkner A, Tiveljunglindell A (2007). Human bocavirus and acute wheezing in children. Clin Infect Dis.

[2] Geoffroy MC, Salvetti A (2005). Helper functions required for wild type and recombinant adeno-associated virus growth. Curr Gene Ther.

[3] Kapoor A, Simmonds P, Slikas E, Li L, Bodhidatta L, Sethabutr O, Triki H, Bahri O, Oderinde BS, Baba MM (2010). Human bocaviruses are highly diverse, dispersed, recombination prone, and prevalent inenteric infections. J Infect Dis.

[4] Allander T, Emerson SU, Engle RE, Purcell RH, Bukh J (2009). A virus discovery method incorporating DNase treatment and its application to the identification of two bovine parvovirus species. Proc Natl Acad Sci U S A.

[5] Inaba Y, Kurogi H, Omori T, Matumoto M (1973). A new serotype of bovine parvovirus. Jpn J Microbiol.

[6] Feng L (2007). Quantification of human bocavirus in lower respiratory tract infections in China. Infect Agent Cancer.

[7] Brown KE, Green SW, Young NS (1995). Goose parvovirus--an autonomous member of the dependovirus genus?. Virology.

[8] Decaro N, Buonavoglia C (2012). Canine parvovirus—A review of epidemiological and diagnostic aspects, with emphasis on type 2c. Vet Microbiol.

[9] Kailasan S, Halder S, Gurda B, Bladek H, Chipman PR, Mckenna R, Brown K, Agbandjemckenna M (2014). Structure of an enteric pathogen, bovine parvovirus. J Virol.

[10] Cotmore SF, Tattersall P (1987). The autonomously replicating parvoviruses of vertebrates. Adv Virus Res.

[11] Wang XS, Ponnazhagan S, Srivastava A (1996). Rescue and replication of adeno-associated virus type 2 as well as vector DNA sequences from recombinant plasmids containing deletions in the viral inverted terminal repeats: selective encapsidation of viral genomes in progeny virions. J Virol.

[12] Babkin IV, Tyumentsev AI, Tikunov AY, Zhirakovskaia EV, Netesov SV, Tikunova NV (2015). A study of the human bocavirus replicative genome structures. Virus Res.

[13] Jiang YH, Xiao CT, Yin SH, Gerber PF, Halbur PG, Opriessnig T (2014). High prevalence and genetic diversity of porcine bocaviruses in pigs in the USA, and identification of multiple novel porcine bocaviruses. J Gen Virol.

[14] Legendre D, Rommelaere J (1994). Targeting of promoters for trans activation by a carboxy-terminal domain of the NS-1 protein of the parvovirus minute virus of mice. J Virol.

[15] Poole BD, Zhou J, Grote A, Schiffenbauer A, Naides SJ (2006). Apoptosis of liver-derived cells induced by parvovirus B19 nonstructural protein. J Virol.

[16] Tullis GE, Burger LR, Pintel DJ (1993). The minor capsid protein VP1 of the autonomous parvovirus minute virus of mice is dispensable for encapsidation of progeny single-stranded DNA but is required for infectivity. J Virol.

[17] Zádori Z, Szelei J, Lacoste MC, Li Y, Gariépy S, Raymond P, Allaire M, Nabi IR, Tijssen P (2001). A viral phospholipase A2 is required for parvovirus infectivity. Dev Cell.

[18] Girod A, Wobus CE, Zádori Z, Ried M, Leike K, Tijssen P, Kleinschmidt JA, Hallek M (2002). The VP1 capsid protein of adeno-associated virus type 2 is carrying a phospholipase A2 domain required for virus infectivity. J Gen Virol.

[19] Lombardo E, Ramírez JC, Garcia J, Almendral JM (2002). Complementary roles of multiple nuclear targeting signals in the capsid proteins of the parvovirus minute virus of mice during assembly and onset of infection. J Virol.

[20] Briassouli A, Kompatsiaris I (1999). Nuclear transport of the major capsid proteins is essential for adeno-associated virus capsid formation. J Virol.

[21] Macara IG (2001). Transport into and out of the Nucleus. Microbiol Mol Biol Rev.

[22] Abaitua F, Hollinshead M, Bolstad M, Crump CM, O'Hare P (2012). A Nuclear localization signal in herpesvirus protein VP1-2 is essential for infection via capsid routing to the nuclear pore. J Virol.

[23] Hennig T, Abaitua F, O'Hare P (2014). Functional analysis of nuclear localization signals in VP1-2 homologues from all herpesvirus subfamilies. J Virol.

[24] Xiang QW, Zou JF, Wang X, Sun YN, Gao JM, Xie ZJ, Wang Y, Zhu YL, Jiang SJ (2013). Identification of two functional nuclear localization signals in the capsid protein of duck circovirus. Virology.

[25] Liu Q, Tikoo SK, Babiuk LA (2001). Nuclear localization of the ORF2 protein encoded by porcine circovirus type 2. Virology.

[26] Walker MP, Lipkin WI (2002). Characterization of the nuclear localization signal of the borna disease virus polymerase. J Virol.

[27] Cros JF, García-Sastre A, Palese P (2005). An unconventional NLS is critical for the nuclear import of the influenza A virus Nucleoprotein and ribonucleoprotein. Traffic.

[28] Grieger JC, Snowdy S, Samulski RJ (2006). Separate basic region motifs within the adeno-associated virus capsid proteins are essential for infectivity and assembly. J Virol.

[29] Ruffing M, Zentgraf H, Kleinschmidt JA (1992). Assembly of viruslike particles by recombinant structural proteins of adeno-associated virus type 2 in insect cells. J Virol.

[30] Wu P, Xiao W, Conlon T, Hughes J, Agbandjemckenna M, Ferkol T, Flotte T, Muzyczka N (2000). Mutational analysis of the adeno-associated virus type 2 (AAV2) capsid gene and construction of AAV2 vectors with altered tropism. J Virol.

[31] Vihinen-Ranta M, Kakkola L, Kalela A, Vilja P, Vuento M (1997). Characterization of a nuclear localization signal of canine parvovirus capsid proteins. Eur J Biochem.

[32] Vihinenranta M, Wang D, Weichert WS, Parrish CR (2002). The VP1 N-terminal sequence of canine parvovirus affects nuclear transport of capsids and efficient cell infection. J Virol.

[33] Lombardo E, Ramírez JC, Agbandjemckenna M, Almendral JM (2000). A beta-stranded motif drives capsid protein oligomers of the parvovirus minute virus of mice into the nucleus for viral assembly. J Virol.

[34] Nüesch JP, Tattersall P (1993). Nuclear targeting of the parvoviral replicator molecule NS1: evidence for self-association prior to nuclear transport. Virology.

[35] Boisvert M, Fernandes S, Tijssen P (2014). Classic nuclear localization signals and a novel nuclear localization motif are required for nuclear transport of porcine parvovirus capsid proteins. J Virol.

[36] Pillet S, Annan Z, Fichelson S, Morinet FR (2003). Identification of a nonconventional motif necessary for the nuclear import of the human parvovirus B19 major capsid protein (VP2). Virology.

[37] Zou W, Cheng F, Shen W, Engelhardt JF, Yan Z, Qiu J (2016). Nonstructural protein NP1 of human bocavirus 1 plays a critical role in the expression of viral capsid proteins. J Virol.

[38] Li Q, Zhang Z, Zheng Z, Ke X, Luo H, Hu Q, Wang H (2013). Identification and characterization of complex dual nuclear localization signals in human bocavirus NPl. J Gen Virol.

[39] Zhang R, Fang L, Cai K, Zeng S, Wu W, An K, Chen H, Xiao S (2016). Differential contributions of porcine bocavirus NP1 protein N- and C-terminal regions to its nuclear localization and immune regulation. J Gen Virol.

